# Correction to “Fabrication of NIR-II-Responsive Polydextran Aldehyde/Gelatin/Gold Nanoparticle-Decorated Graphene Oxide Nanocomposite Hydrogels for Antibacterial Applications”

**DOI:** 10.1021/acsapm.6c01120

**Published:** 2026-04-27

**Authors:** Xi-Er Chen, Chen-Jie Yan, Cheng-Hsun Lu, Yi-Cheun Yeh

The original paper should have been included in the Special Issue Early Career Forum 2024: https://pubs.acs.org/toc/aapmcd/6/23.

